# Time-, Sex-, and Dose-Dependent Alterations of the Gut Microbiota by Consumption of Dietary Daikenchuto (TU-100)

**DOI:** 10.1155/2018/7415975

**Published:** 2018-02-22

**Authors:** Jun Miyoshi, Kentaro Nobutani, Mark W. Musch, Daina L. Ringus, Nathaniel A. Hubert, Masahiro Yamamoto, Yoshio Kase, Mitsue Nishiyama, Eugene B. Chang

**Affiliations:** ^1^Department of Medicine, Knapp Center for Biomedical Center, The University of Chicago, Chicago, IL, USA; ^2^Tsumura Research Laboratories, Tsumura & Co., Ami, Ibaraki, Japan

## Abstract

Medications or dietary components can affect both the host and the host's gut microbiota. Changes in the microbiota may influence medication efficacy and interactions. Daikenchuto (TU-100), a herbal medication, comprised of ginger, ginseng, and Japanese pepper, is widely used in Japanese traditional Kampo medicine for intestinal motility and postoperative paralytic ileus. We previously showed in mice that consumption of TU-100 for 4 weeks changed the gut microbiota and increased bioavailability of bacterial ginsenoside metabolites. Since TU-100 is prescribed in humans for months to years, we examined the time- and sex-dependent effects of TU-100 on mouse gut microbiota. Oral administration of 1.5% TU-100 for 24 weeks caused more pronounced changes in gut microbiota in female than in male mice. Changes in both sexes largely reverted to baseline upon TU-100 withdrawal. Effects were time and dose dependent. The microbial profiles reverted to baseline within 4 weeks after withdrawal of 0.75% TU-100 but were sustained after withdrawal of 3% TU-100. In summary, dietary TU-100 changed mouse microbiota in a time-, sex-, and dose-dependent manner. These findings may be taken into consideration when determining optimizing dose for conditions of human health and disease with the consideration of differences in composition and response of the human intestinal microbiota.

## 1. Introduction

The gut microbiota has a systemic effect on human health [1, 2]. Most ingested compounds, be they dietary, taken for therapeutic benefits, or taken for other purposes, influence the microbiota and conversely the microbiota can also metabolize many orally ingested substances. Thus bacterial enzymes, involved in bacterial metabolism, can also serve to inactivate or activate drugs or herbal medications ingested by the host. Examples of this include the bacterial cytochrome encoding operon of* Eggerthella lenta* that metabolizes digoxin [3], bacterial glucuronidases that are responsible for the reactivation of the phase II product of the chemotherapeutic drug irinotecan [4], and bacterial glycosidases, and glucosidases that remove sugars, altering intestinal absorption and activity of ginseng dammarane saponins, including ginsenoside Rb1 [5]. Therefore it is important that the bidirectional interactions between medications and intestinal microbiota are considered when assessing drug pharmacokinetics and clinical efficacy [6, 7]. The ability of compounds in TU-100 to alter the microbiota may be due to the presentation of substrates, providing a survival benefit for these bacteria as they may metabolize. However, we cannot exclude the possibility that some compounds in TU-100 inhibit the growth of some bacteria. Both ginger and ginseng extracts as well as shogaols from sansho pepper have been demonstrated to have antimicrobial action [8–11] but the predominant intestinal bacteria were not included in these studies. Additionally, these studies were performed in vitro, considerably different from conditions in the intestinal tract.

Daikenchuto (TU-100) is a herbal medication used under the aegis of the traditional Kampo therapeutic system in Japan. Kampo has been used for over a thousand years and TU-100 is widely used in Japan to improve gastrointestinal (GI) motility, especially in the lower GI tract, including prevention of postoperative paralytic ileus. TU-100 is an aqueous extract from a mixture of 50% ginger* (Zingiber official)*, 20% Japanese pepper* (Zanthoxylum piperitum),* and 30% ginseng* (Panax ginseng)*, prepared according to an established formulation [12]. Several clinical studies including multicenter randomized clinical trials have been performed to demonstrate the efficacy of TU-100 and have shown that the administration of TU-100 improves postoperative bowel function [13–17].

Although it was approved in 1986 by the Japanese government as a pharmaceutical compound and various mechanisms to explain the effect of TU-100 on improving GI tract motility and blood flow have been proposed [18–24], the mechanisms underlying TU-100's actions are not completely understood. A variety of compounds in TU-100 may lead to complementary and synergetic effects. Ginseng contains many complex carbohydrates and dammarane saponin ginsenosides. Some of these are metabolized and converted to bioactive metabolites by gut microbiota, which makes them more rapidly absorbable. We published that, in mice, dietary administration of TU-100 for 4 weeks altered gut microbiota and increased the ability of the microbiota to convert a major ginsenoside (Rb1) to bioactive compound K [25]. Gingerols and shogaols in ginger are rapidly absorbed in the small intestine and are involved in the enterohepatic circulation via metabolism by the liver, intestine, and gut microbiota [26]. It is well known that gut microbiota plays a role in many GI tract functions of the host including motility [27, 28] and xenobiotic metabolism [29, 30]. Also noteworthy is that sex-specific differences have been reported both with respect to the gut microbiota [31, 32] and also with respect to drug metabolism [33]. We recently reported that long-term consumption of TU-100 affects murine hepatic and intestinal drug metabolizing enzymes [34]. Collectively these findings suggest that the gut microbiota can mediate some of the actions of TU-100 and that more information is needed to understand how greater efficacy and dosing of TU-100 can be achieved.

Since TU-100 can be prescribed long term in clinical settings, to understand its mechanisms of action, it is important to delineate TU-100's effects in terms of long-term exposure, dose dependency, sex specificity, and reversibility of the effects after cessation of TU-100 exposure in an in vivo model. Therefore, we undertook a long-term, 24-week study, to investigate the chronological change of the gut microbiota in the presence and absence of TU-100 administration, its dose dependence on TU-100, and its reversibility after withdrawal of TU-100 in female and male mice. We believe that this is the first comprehensive study to examine the effect of a herbal medication on the gut microbiota.

## 2. Materials and Methods

### 2.1. Animals

C57Bl/6J mice were bred in-house at the University of Chicago Animal Care Facilities (Institutional Animal Care and Use Committee Protocol 71084). Five breeding pairs were set up when mice were 7–9 weeks old and called the F_0_ generation. Their progeny were termed the F_1_ generation and were used to set up 25 breeding pairs, with care being taken to avoid using littermates for any breeding pair. For the present study, mice of the F_2_ generation were used. The F_2_ generation pups were weaned at 21 days after birth and ear tagged for identification. Fresh bedding is provided every 14 days in our animal facility. Between weaning and the start of the experiments (see below), to decrease cage-cage variability, a mixed bedding protocol was implemented. Thus, 3-4 days and also at 8–10 days after fresh bedding was provided, the mouse bedding was removed and mixed. The bedding from all cages of females and male animals was mixed together and the mixed bedding was distributed back to all cages. Mice were maintained on a 12-hour light 12-hour dark cycle, with light initiated at 6 AM.

### 2.2. Diet and Drug Treatment

Mice were fed Teklad Global 18% Protein Rodent Diet (2018) (Envigo, Madison, WI, USA) until one week before the start of experiments and then switched to the defined, AIN-76A Purified Diet (CA. 170481) (The American Institute of Nutrition, 1977) (Envigo) for one week. TU-100 was obtained as a powder from Tsumura & Co. (Ami, Ibaraki, Japan). TU-100 was included at 0.75, 1.5, or 3% wt/wt in AIN76A (Institutional Animal Care and Use Committee protocol 72101). The dosage of TU-100 for murine experiments was based on previous reports that aimed to achieve blood concentrations of major TU-100 compounds, similar to that reported in human data [24–26, 35, 36].

### 2.3. Study Designs

**Figure 1:**
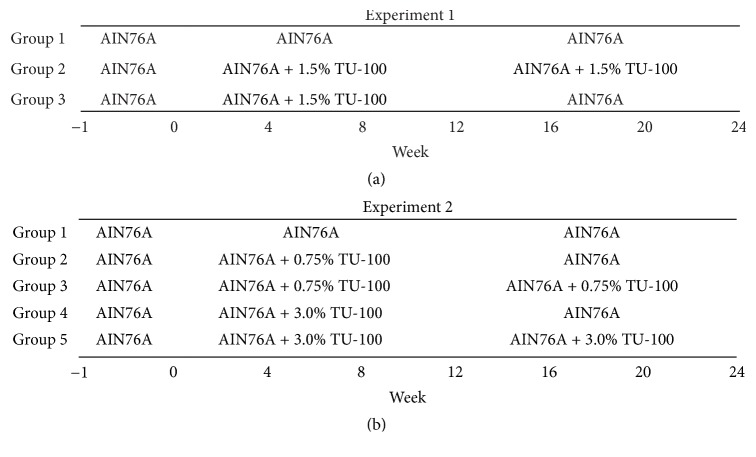
*Timelines of Dietary TU-100 Administration*. All mice were fed AIN76-A for one week before starting experiments. (a) Forty-eight females and 48 males were assigned to 3 groups (groups 1–3). Mice in group 1 and group 2 were fed AIN76-A and 1.5% TU-100 for 24 weeks, respectively. Group 3 was fed 1.5% TU-100 for 12 weeks and then returned to AIN-76A for another 12 weeks. (b) Forty-five males were stratified into 5 groups (groups 1–5). Group 1 was 5 mice and other groups were 10 mice. Group 1 were fed AIN-76A for 24 weeks. Group 2 and group 4 were fed 0.75% and 3.0% TU-100 for 12 weeks and then returned to AIN-76A for another 12 weeks, respectively. Group 3 and group 5 were fed 0.75% and 3.0% TU-100 for 24 weeks, respectively.

Forty-eight female and 48 male mice were used for the first experiment and were 7–12 weeks old at the start of the experimental protocol, labeled as “week 0.” Mice were assigned to 3 groups (groups 1–3) and each group contained 16 female and 16 male mice. Mice in group 1 and group 2 were fed AIN-76A and 1.5% TU-100 for 24 weeks (weeks 0–24), respectively. Group 3 was fed 1.5% TU-100 for 12 weeks (weeks 0–12) and then returned to AIN-76A for another 12 weeks (weeks 12–24). Half of the mice in each group were sacrificed at week 12 and the remaining mice at week 24 (Figure 1(a)). In the second experiment, to study dose-dependent effects, 45 male mice were used and mice were 6–9 weeks old at week 0. The mice were stratified into 5 groups (groups 1–5). Group 1 (5 mice) were fed AIN-76A for 24 weeks (weeks 0–24). Group 2 and group 4 (10 mice in each group) were fed 0.75% and 3.0% TU-100 for 12 weeks (weeks 0–12) and then returned to AIN-76A for another 12 weeks (weeks 12–24), respectively. Group 3 and group 5 (10 mice in each group) were fed 0.75% and 3.0% TU-100 for 24 weeks (weeks 0–24), respectively. Half of the mice in groups 2–5 were sacrificed for tissue samples at week 12 and all mice in group 1 and the other half in groups 2–5 were harvested at week 24 (Figure 1(b)).

### 2.4. Fecal Samples

Fecal pellets were harvested from the mice every 4 weeks from week 0 through week 24 (Figure 1). The stool was always taken at 6 AM as mouse fecal microbiota changes in a circadian manner [37].

### 2.5. DNA Extraction and 16S rRNA Gene Sequencing Analysis

DNA was extracted from stool pellets by standard, published protocols [38]. Sequences were obtained at the Next Generation Sequencing Core in the Biosciences Division at Argonne National Laboratory with standard protocols (http://www.earthmicrobiome.org/protocols-and-standards/16s/) using MiSeq (Illumina, San Diego, CA, USA) for sequencing. DNA sequences were analyzed by Quantitative Insights into Microbial Ecology (QIIME) version 1.9.1 [39]. Operational taxonomic units (OTUs) were picked up at 97% sequence identity using the GreenGenes Database (http://greengenes.secondgenome.com/).

### 2.6. Statistical Analysis

Mann–Whitney* U* test was performed with GraphPad Prism (GraphPad Software, La Jolla, CA, USA).* U* tests were employed to examine the changes of the proportions of phyla and genera after TU-100 administration. Kruskal-Wallis test was performed on QIIME generated bacterial abundance percentages to compare the abundance of each OTU between groups. *P* values less than 0.05 were considered statistically significant.

## 3. Results and Discussion

### 3.1. Comparison of Fecal Microbiota between Sexes before Administration of TU-100

**Figure 2:**
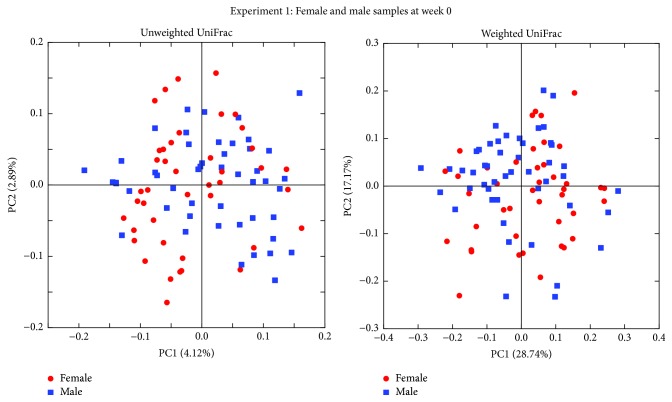
*Sex dependence of the fecal microbiota*. PCoA plots of fecal microbiotas of female (red circle) and male (blue square) mice at week 0 in experiment 1. PCoA plots of both unweighted and weighted UniFrac distances demonstrate no difference between sexes. Forty-eight female and 48 male mice were used for the experiment; however, 6 female mice samples were excluded as less than 5000 sequences were obtained.

Analysis of fecal microbiota was performed before switching mice to a defined diet to determine the variability of the starting population. 16S rRNA genes were sequenced and principal coordinate analysis (PCoA) plots generated by QIIME from MiSeq data for 48 female and 48 male samples. Since 6 samples from female mice gave less than 5000 sequences, these samples were excluded. The PCoA plots showed the similar distribution between sexes in both unweighted and weighted UniFrac distances (Figure 2). Unweighted UniFrac distances describe the presence or absence of OTUs in samples while weighted UniFrac distances describe the distribution of proportions of OTUs in samples.

### 3.2. Difference of Fecal Microbiota with Different Administration Pattern of TU-100

**Figure 3:**
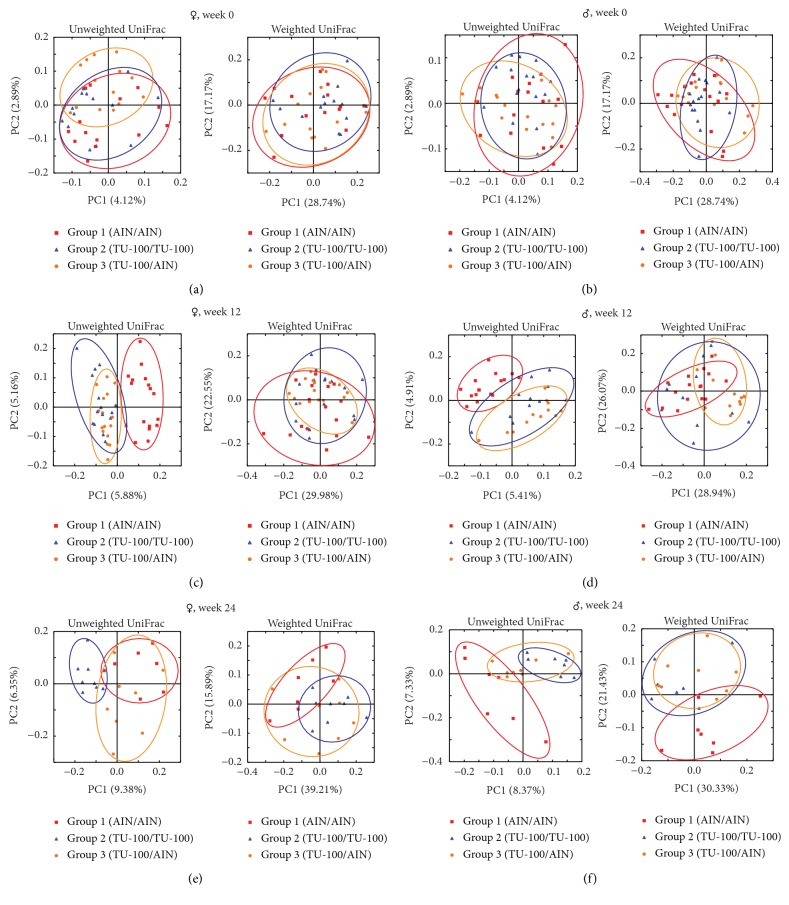
*Dietary 1.5% TU-100 Alters Fecal Microbiota and the Effect is Reversible*. PCoA plots of unweighted and weighted UniFrac distances were generated for female ((a), (c), and (e)) and male ((b), (d), and (f)) samples at weeks 0 ((a) and (b)), 12 ((c) and (d)), and 24 ((e) and (f)). Red squares indicate group 1 (fed AIN76A diet for 24 weeks), blue triangles group 2 (fed TU-100 for 24 weeks), and orange circles group 3 (fed TU-100 for 12 weeks and then AIN-76A for 12 weeks). ((a) and (b)) At week 0, the microbiota among mice in each group appeared to be randomly distributed in each sex. ((c) and (d)) At week 12, plots for mice in group 2 and group 3 after 12-week consumption of 1.5% TU-100 clustered together and separately from group 1 without TU-100 in unweighted UniFrac distances for both sexes. ((e) and (f)) At week 24, the plots of group 3 appeared to be between groups 1 and 2 in PCoA of unweighted UniFrac distances for both sexes.

Mice were placed into 3 groups (groups 1–3) in each sex of littermates. As shown in Figure 1(a), mice in group 1 and group 2 were fed AIN76-A and 1.5% TU-100 for 24 weeks (weeks 0–24), respectively. Mice in group 3 were fed 1.5% TU-100 for 12 weeks (weeks 0–12) and then switched to AIN-76A for another 12 weeks (weeks 12–24). In PCoA plots at week 0, the microbiota among mice in each group in each sex appeared to be randomly distributed (Figures 3(a) and 3(b)) before the start of TU-100 administration. After 12 weeks, data for mice in group 2 and group 3 (fed dietary TU-100) clustered together and separately from group 1 without TU-100. Clustering of TU-100 fed mice was observed for both male and female mice in unweighted UniFrac distances. However, differences were not observed in weighted UniFrac distances (Figures 3(c) and 3(d)). These findings demonstrate that dietary TU-100 for 12 weeks changes the bacterial lineages in fecal samples of both sexes; however, the proportion of bacterial lineages was not dramatically changed. At 24 weeks, the plots of group 3 appeared to be separated from the plots of not only group 1 but also group 2 and to be plotted between groups 1 and 2 in both female and male samples in PCoA of unweighted UniFrac distances (Figures 3(e) and 3(f)). This result demonstrated the reversibility of the fecal microbiota at 12 weeks after withdrawal of 12-week intake of 1.5% TU-100 in both sexes. In addition, PCoAs of both unweighted and weighted UniFrac distances indicated that 24-week 1.5% TU-100 consumption changed the composition of the fecal microbiota.

### 3.3. Chronological Change of Fecal Microbiota with TU-100

**Figure 4:**
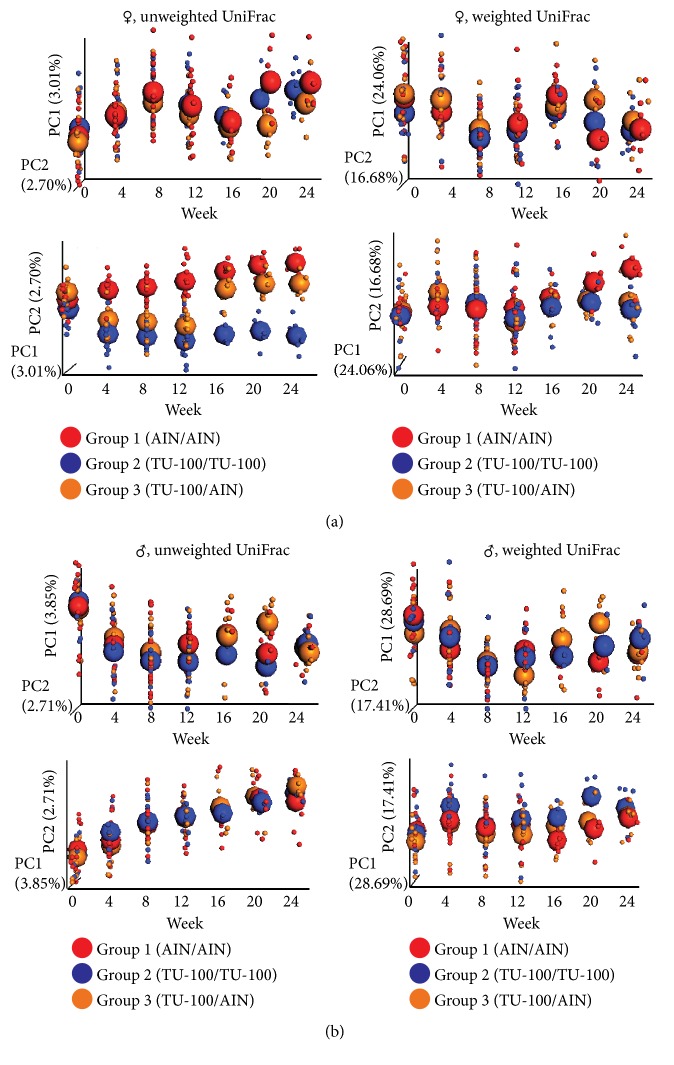
*Chronological Changes of Fecal Microbiotas by 1.5% TU-100*. Female and male fecal microbiotas were analyzed every 4 weeks from weeks 0 to 24. PCoA plots of unweighted UniFrac distances and weighted UniFrac distances over time are shown for female (a) and male (b) samples. Small dots represent individual samples and large spheroids represent means of each group. Red, blue, and orange dots/spheroids are for group 1 (fed AIN-76A for 24 weeks), group 2 (fed 1.5% TU-100 for 24 weeks), and group 3 (fed 1.5% TU-100 for 12 weeks and then AIN-76A for 12 weeks), respectively.

To determine how quickly the microbiota changes after addition to the diet and how quickly the changes are reversible, samples were analyzed from fecal DNA at weeks 0, 4, 8, 12, 16, 20, and 24 and the data are shown in Figure 4. In group 1 that never received dietary TU-100, changes in microbiota composition mostly likely reflect the effects of aging. Thus, in interpreting changes with and without TU-100, these data should be viewed in the context of changes that occur with aging. For group 2 which received dietary TU-100 for the entire 24 weeks, changes were nearly complete by week 4 and showed some variation after this point. For group 3, they received dietary TU-100 for weeks 0–12 and then diet without TU-100 for weeks 12–24; as for group 2, changes with TU-100 were nearly complete within 4–8 weeks. Upon removal of TU-100 at week 12, a near-complete reversal was observed at week 16. These chronological changes were more apparent in PCoA of unweighted UniFrac distances of female samples (Figure 4(a)) than male samples (Figure 4(b)). PC1 and PC2 axes are shown for PCoA plots in each panel.

### 3.4. Alteration of Bacterial Phyla and Genera with TU-100 Administration

**Figure 5:**
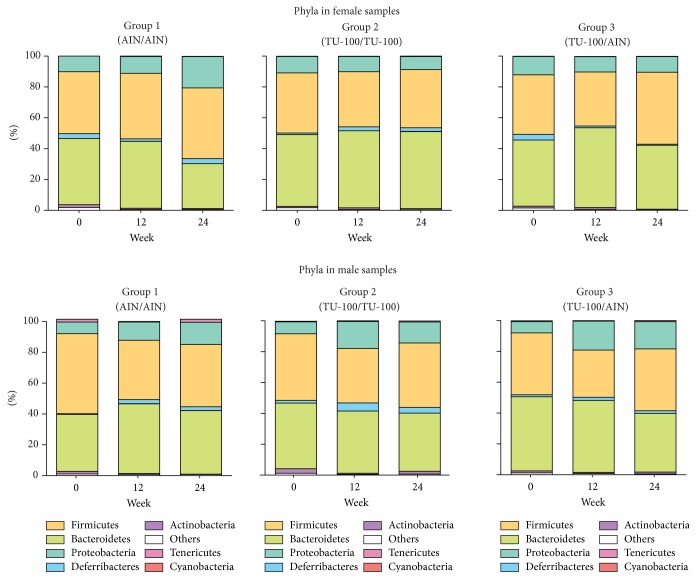
*Dietary 1.5% TU-100 Alters the Phylum Compositions*. Bacterial taxonomy was designated by QIIME based on 16S rRNA sequences. Phyla are listed in order of percentage of the population from greatest to least abundance.

16S rRNA sequences were next analyzed by QIIME for taxonomy assignment. We analyzed the composition of phyla in each group at weeks 0, 12, and 24 of female and male samples (Figure 5). Phyla with more than 1.0% of the population in any group at week 24 are listed in Table 1. In the samples from female mice, Bacteroidetes was increased and Proteobacteria was decreased significantly in the comparison of group 1 and group 2. On the other hand, in males, these phyla did not show significant changes. In male samples, however, the phylum Actinobacteria showed a significant increase even though the percentage of the population was still low. Next, we determined the representation of various genera in the same samples.  Table 2 shows genera with more than 1.0% of the population in any group that are significantly different between group 1 and group 2 at week 24. In female samples, the genus* Bacteroides*, one of the family Rikenellaceae, one of the family S24-7,* Turicibacter,* and one of the family Clostridiaceae increased with TU-100 administration for 24 weeks. In contrast, over the same treatment, one genus of the order Clostridiales and one genus of the family Desulfovibrionaceae decreased significantly. On the other hand, in male samples, one genus of the family Coriobacteriaceae and the genus* Allobaculum* increased and the genera* Turicibacter* and* Ruminococcus* decreased significantly. Furthermore, we examined the change of specific OTUs and the top 20 OTUs that demonstrated significant increases or decreases are shown in Supplementary Table 1 (listed in order of* P* value). These results showed that 24-week intake of 1.5% TU-100 altered the fecal microbiota in both sexes but the specific changes at the level of phylum, genus, and OTU were different between sexes.

### 3.5. Dose-Dependent Effect of TU-100 on Fecal Microbiota

**Figure 6:**
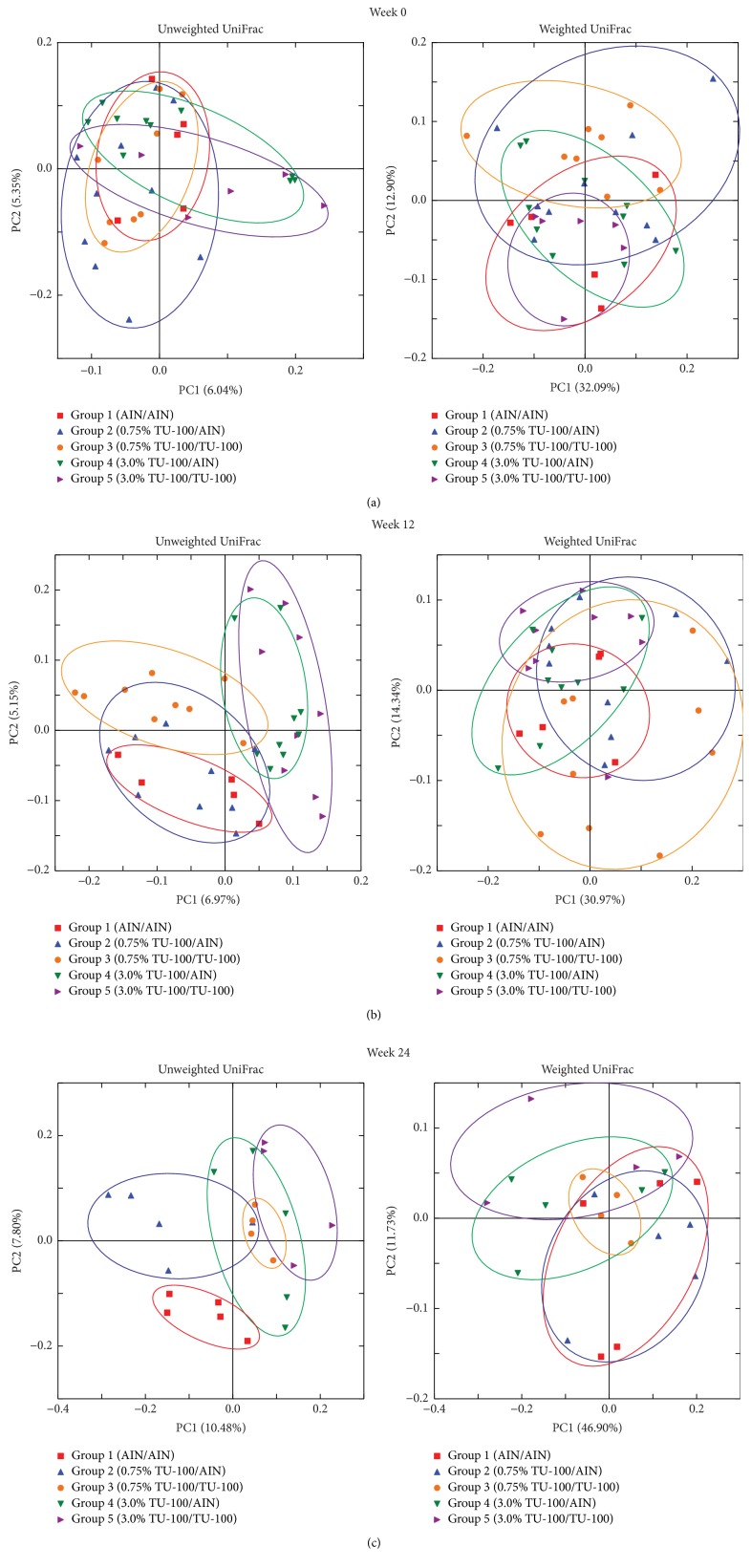
*Dietary TU-100 Alters Fecal Microbiota in a Dose-Dependent Manner*. PCoA plots of unweighted UniFrac distance and weighted UniFrac distances were generated for fecal microbiotas at weeks 0 (a), 12 (b), and 24 (c). Red squares, blue triangles, orange circles, green triangles, and purple triangles indicate groups 1–5, respectively. Groups 1, 3, and 5 were fed AIN-76A and 0.75% and 3.0% TU-100 for 24 weeks, respectively. Groups 2 and 4 were fed 0.75% and 3.0% TU-100 for 12 weeks and then switched to AIN-76A for another 12 weeks, respectively. (a) At week 0, the fecal microbiotas in each group appeared to be randomly distributed. (b) At week 12, the plots of group 1, groups 2 and 3, and groups 4 and 5 appeared to be clustered, respectively, in PCoAs of unweighted UniFrac distances. (c) At week 24, the plots of groups 3, 4, and 5 appeared to be clustered. Group 2 was separated from group 3 and seemed to show similar distribution to group 1 in PCoAs of both unweighted and weighted UniFrac distances.

To determine if the effects of dietary TU-100 are dose dependent, diets were prepared with half and twice the TU-100 (0.75 and 3% wt/wt) as used in experiment 1. For this experiment, 5 groups of male mice were used as shown in Figure 1(b). For practical considerations of the numbers of animals to be handled in each experiment, these studies focused only on male mice. Groups 1, 3, and 5 were fed AIN-76A and 0.75% and 3.0% TU-100 for 24 weeks (weeks 0–24), respectively. Groups 2 and 4 were fed 0.75% and 3.0% TU-100 for 12 weeks (weeks 0–12) and then switched to AIN-76A for another 12 weeks (weeks 12–24), respectively. The fecal microbiotas in each group appeared to be randomly distributed in PCoA plots at week 0 (Figure 6(a)). At week 12, the plots of group 1 (AIN76-A), groups 2 and 3 (0.75% TU-100), and groups 4 and 5 (3.0% TU-100) appeared to be clustered, respectively, in PCoAs of unweighted UniFrac distances while the clustering of the plots of groups 2 and 3 was less apparent than groups 4 and 5 (Figure 6(b)). These results indicated that TU-100 administered for 12 weeks can alter the fecal microbiota at both concentrations of 0.75% and 3.0% while the extent of change to the microbiota caused by each concentration differs. At week 24, the plots of groups 3, 4, and 5 appeared to be clustered and group 2 was separated from group 3 in PCoAs of both unweighted and weighted UniFrac distances. Group 2 also seemed to show the similar distribution to group 1 rather than to group 3 (Figure 6(c)). These findings can be summarized by the following: (a) the administration of 0.75% TU-100 for 24 weeks caused similar changes in fecal microbiota as 3.0% TU-100 for 24 weeks and (b) the fecal microbiota of animals, which had been administered 0.75% TU-100 for 12 weeks and then maintained in the absence of TU-100 for 12 weeks, reverted to a baseline profile, whereas (c) those that had been administered 3.0% TU-100 for 12 weeks and then maintained in the absence of TU-100 for 12 weeks showed no reversibility in profile of the fecal microbiota. Of note the group size for these dose-dependence experiments is smaller (5 versus 7-8 for experiment 1). Statistical analysis of data from smaller group sizes will determine statistical significance only when larger changes are observed in the smaller groups. Smaller changes that might be significant in an experiment with larger group sizes will not be found to be significant in experiments with smaller group sizes.

### 3.6. Chronological Change of Fecal Microbiota with Different Dose TU-100

**Figure 7:**
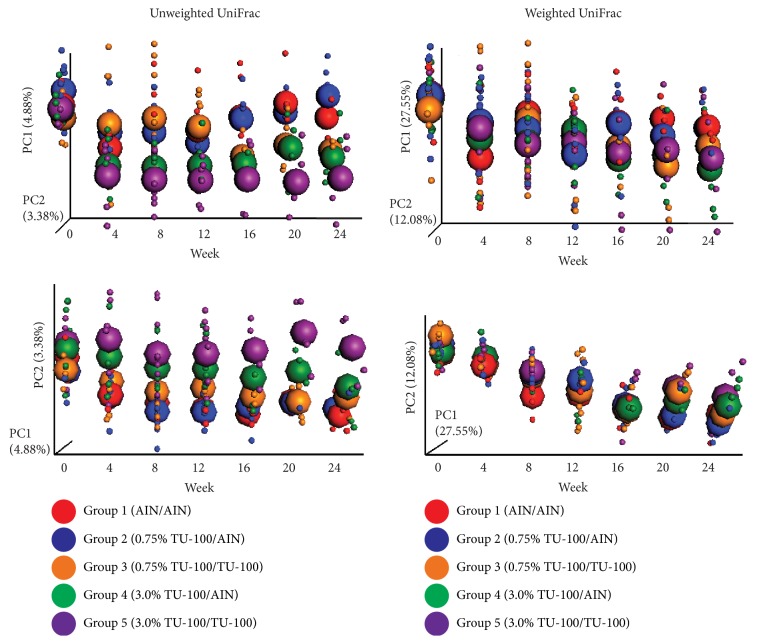
*Dose-Dependent Chronological Changes of Fecal Microbiotas by TU-100*. Fecal microbiotas were analyzed every 4 weeks from weeks 0 to 24. PCoA plots of unweighted UniFrac distances and weighted UniFrac distances over time are shown. Small dots represent individual samples and large spheroids represent means of each group. Red, blue, orange, green, and purple dots/spheroids are for group 1 (fed AIN-76A for 24 weeks), group 2 (fed 0.75% TU-100 for 12 weeks and then AIN-76-A for 12 weeks), group 3 (fed 0.75% for 24 weeks), group 4 (fed 3.0% TU-100 for 12 weeks and then AIN-76-A for 12 weeks), and group 5 (fed 3.0% for 24 weeks), respectively.

We next analyzed the chronological change of fecal microbiota in each group using fecal DNA samples at weeks 0, 4, 8, 12, 16, 20, and 24 (Figure 7). The continuous changes in gut microbiome observed in group 1 (AIN-76A for 24 weeks) are likely related to age-dependent changes in gut microbiota. A diet containing 3.0% TU-100 resulted in a microbiota that had shifted by week 4 (groups 4 and 5). A diet containing 0.75% TU-100 changed the microbiota less than a diet containing 3.0% TU-100 at week 12, while continuous consumption of 0.75% TU-100 for longer than 16 weeks showed changes of microbiota similar to those caused by 3.0% TU-100 (group 3). Withdrawal of 0.75% TU-100, after consumption for 12 weeks, demonstrated that the reversibility of microbiota was complete by week 16 (i.e., 4 weeks after withdrawal of 0.75%, group 2). However there was no reversibility in the microbiota after withdrawal of 3.0% TU-100 (group 4). These findings indicate that (1) TU-100 has a dose-dependent effect, (2) the effect of 0.75% TU-100 on fecal microbiota is reversible, and (3) the administration of 3.0% TU-100 for 12 weeks has a long-lasting influence on the fecal microbiota.

### 3.7. Alteration of Bacterial Phyla and Genera with Different Dose TU-100 Administration

**Figure 8:**
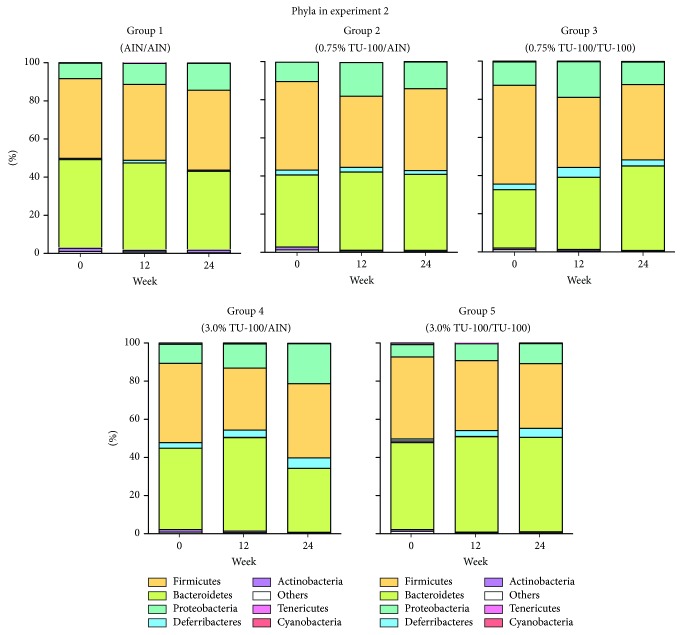
*Dietary 0.75/3.0% TU-100 Alters Phylum Compositions*. Bacterial taxonomy was designated for each group at weeks 0, 12, and 24 as in experiment 1. Phyla are listed in order of percentage of the population from greatest to least abundance.

We examined the gut microbial composition in each group at weeks 0, 12, and 24 (Figure 8). The phyla with more than 1.0% of the population in any group are listed in Table 3. The phylum Actinobacteria decreased significantly in group 3 compared to group 1. Although group 5 did not present any significant difference from group 1, groups 3 and 5 demonstrated the same trend of change at the level of phylum.  Table 4 shows the genera with more than 1.0% of the population in any group showing a significant difference between group 1 and group 3 or group 1 and group 5. In the comparison between group 1 and group 3, the genera one of the family Rikenellaceae,* Rikenella* and* Lactococcus* increased while one genus of the family Coriobacteriaceae decreased significantly in group 3. On the other hand, group 5 showed the significant increase of one genus of the family Rikenellaceae and* AF12* and decrease of one genus of the family Coriobacteriaceae and* Ruminococcus*, compared to group 1. Of note, between 0.75% and 3.0% TU-100, genera that showed significant changes with one dose of TU-100 also demonstrated the same trend of change with the other dose. We also analyzed the change of OTUs in the comparison between group 1 and group 3 and group 1 and group 5. Supplementary Table 2 shows the top 20 OTUs that increased significantly and all 15 OTUs that decreased significantly in group 3 compared to group 1. The top 20 OTUs that increased and decreased significantly in the comparison between group 1 and group 5 are shown in Supplementary Table 3. These OTUs are ordered by *P* value. These results demonstrated that (1) both 24-week consumption of 0.75% and 3.0% TU-100 changed the fecal microbiota, (2) these changes differed between doses although they elicited a similar trend at the level of phylum and genus, and (3) withdrawal of 3.0% TU-100 after 12-week administration did not show reversibility of microbiota even after a 12-week withdrawal period.

The present study demonstrates the effect of TU-100 administration and withdrawal on fecal microbiota in both female and male mice and the effects of different doses in male mice for 24 weeks. Our study provides insight into determining the clinical efficacy and safety of TU-100 and its influence on the microbiota by examining the following parameters: (1) both female and male animals were studied, (2) chronological changes over a long (24 weeks) observation period, (3) analyses of reversibility of microbiota after cessation of TU-100, and (4) analyses of the dose-dependent effects of TU-100. It is well known that the intestinal microbiota in mice differs from humans. TU-100 may alter the intestinal microbiota by providing substrates for certain bacterial species, increasing their abundance, or some components may provide compounds with antimicrobial actions against certain intestinal bacteria. It is not possible to predict what changes might occur in the human intestinal microbiota from changes in phyla and genera that changed in mice. However, if the bacteria have similar properties in the human and mouse microbiota, changes may occur and remained to be determined.

A general consideration of microbiota studies is to minimize variations in the starting microbiota between groups prior to experimentation, that is, to start with a consistent baseline. It is increasingly recognized that, even within the same facility, and using the same mouse strain, there are room to room and cage to cage differences that influence the microbial profile. For this purpose, in the present study, we adopted a protocol of mixed bedding among all cages in each experiment from weaning until the start of the experiments as described in the Materials and Methods. The plots in each group appeared to be randomly distributed in PCoAs at the start point among each sex and experiment. We attribute this to our methodology of bedding transfers. The mixed bedding protocol is thought to reduce the variability of fecal microbiota among cages safely (Miyoshi et al., manuscript in preparation).

In the present study, the doses of TU-100 were determined to achieve blood concentrations of major TU-100 compounds similar to those in human [24–26, 35, 36]. Our first experiment showed the continuous chronological change of fecal microbiota with the administration of 1.5% TU-100 for 24 weeks and the reversibility of fecal microbiota after withdrawal. These results suggest that longer-term consumption of 1.5% TU-100 more effectively alters the composition of gut microbiota compared to shorter treatment periods and that the effect of 1.5% TU-100 on gut microbiota does not persist for a long time after its cessation (Figures 3 and 4). Importantly, the greater effect of TU-100 on female compared to male animals suggests that there are sex differences in response to dietary TU-100. In addition, our second experiment conducted in male animals demonstrated that 0.75% and 3.0% TU-100 lead to similar alterations of fecal microbiotas after 16-week administration, with the changes occurring much faster (near completion in 4 weeks) in the groups fed 3.0% TU-100. A reversibility of changes in the fecal microbiota was observed after withdrawal of 12-week 0.75% TU-100 but not after withdrawal of 12-week 3.0% TU-100. These results show that the higher dose of TU-100 alters the composition of gut microbiota more rapidly and its effects persist over a longer period, compared to a lower dose of TU-100.

Our recent report using the same murine model demonstrated that dietary TU-100 modulates the transcript and protein expression of drug metabolizing enzymes and drug transporters in the liver, small intestine, and colon in a dose- and sex-dependent manners and that in most cases the effects were reversible after cessation of TU-100 treatment [34]. Taken together, our results show an interactive network between consumption of TU-100, alterations of gut microbiota, and changes in host xenobiotic metabolism. This idea is supported by several previous reports that gut microbiota plays an important role in regulating xenobiotic metabolism in the host [30, 40–43]. Interventions that can modify these interactions could be used to improve clinical efficacy and safety of TU-100.

Our analysis of the changes of phyla and genera revealed interesting differences between sexes. Bolnick et al. [31] demonstrated that the diet-microbiota association is sex-dependent and dietary TU-100 may affect gut microbiota differently in female and male animals. We speculate that this sex difference occurs by the synergistic effects of the following: (1) the difference in composition of microbiota between sexes at the start of TU-100 administration, (2) a sex-dependent selection of colonized microbes, and (3) a difference in metabolism/bioactive-conversion of TU-100 in the two sexes. The difference of gut microbiota contributes to the difference of host drug metabolism and therapeutic efficacy [44] and our findings seem to underscore the importance of analyzing both female and male animals for investigating the drug effect on gut microbiota. The change of bacterial compositions in male animals with 1.5% TU-100 was different from our previous study using male animals [25], underscoring the challenges that microbiota studies pose. First one is the length of the observation period (the present study was for 24 weeks and the previous study for 4 weeks). Second, although we used C57Bl/6J house-bred mice for both studies, these studies were conducted at different times (the present study was in 2015 and the previous study in 2011) and each study used a different room in our mouse facility, although they were maintained under the same facility regulations. Several reports have shown that differences in gut microbiota between research facilities can influence the outcome of experiments even in the same strain of mice. For example, Ivanov et al. showed that there is a difference in gut microbiota between mice from Jackson Laboratory and Taconic Farms, leading to a difference in Th17 population in lamina propria of the small intestine [45]. In addition, Moon et al. [46] observed different IgA phenotypes between two facilities in the same strain of mice and found that phenotypes can be vertically transmitted through the microbiota. These findings highlight the importance of using cohorts with normalized starting microbiota. As this was not done for our previous cohorts, differences in starting microbiota likely influenced the results. The present study, however, began with large starting cohort that was normalized for starting microbiota and housed in one specific room. While this made the experiment labor-intensive and technically challenging, reproducibility was enhanced that was conducive to meaningful comparisons.

We therefore addressed the question of whether there was variability between our experiments 1 and 2. In experiment 2, the genus of the family of Rikenellaceae increased with both 0.75% and 3.0% TU-100 and the genus of the family Coriobacteriaceae decreased with both 0.75% and 3.0% TU-100, and some genera showing significant change with one dose of TU-100 presented the same trend of change with the other dose. Taken together, the alterations of the microbiota after 0.75% and 3.0% TU-100 administration for 24 weeks appeared to be similar. On the other hand, the changes of genera with 1.5% TU-100 seemed different from the changes with 0.75% and 3.0% TU-100 (Tables 2 and 4). We speculate that these differences in alterations of genera between 1.5% and 0.75/3.0% TU-100 are due subtle differences in starting microbiota. The plots of experiments 1 and 2 at week 0 clustered separately in both unweighted and weighted UniFrac distances (Supplementary Figure 1(A)) and the bacterial phyla compositions of each experiment differed (Supplementary Figure 1(B)). This difference could be because the experiments were conducted separately, although all animals for the two experiments were derived from the same 25 breeding pairs and housed in the same room. Given our finding of the aging effect on gut microbiota, it is possible that the microbiota to which litters are exposed by their sires and dams can be different along with the aging of breeding pairs.

We also examined the change of 16S rRNA OTUs, recognizing that these data cannot resolve beyond a genus level and provide no functional information. Among females and males in experiment 1, many OTUs that belong to the family of Desulfovibrionaceae decreased and many that belong to the genus* Allobaculum* increased. The family of Desulfovibrionaceae includes the genera* Desulfovibrio*,* Desulfobaculum*,* Desulfocurvus*,* Bilophila*, and* Lawsonia*[47] and the genus* Desulfovibrio* is listed in our Supplementary Table 1. The genera* Desulfovibrio*,* Desulfobaculum,* and* Desulfocurvus* are regarded as sulfate-reducing bacteria [47]. The genus* Allobaculum* contains butyrate-producing members (our group, unpublished), and butyrate is the main energy source for colonocytes [48]. More ideal would have been additional studies using metagenomic, metatranscriptomic, and metabolomic data to assess functional profiles of microbial communities. These studies are planned for the future. Another limitation of the present study is the fact that we used the fecal samples to analyze microbiota that are an admixture of luminal microbiota of small intestine and large intestine, where the primary effects of TU-100 on regional GI tract microbiota could therefore not be determined.

## 4. Conclusions

The administration of dietary TU-100 alters murine fecal microbiota over time for 24 weeks. These changes are time, sex, and dose dependent. At higher doses of TU-100, the changes in gut microbiota are more rapid, pronounced, and sustained. Whether TU-100 changes human intestinal microbiota will determine if similar changes occur.

## Figures and Tables

**Table 1:** Phyla with more than 1.0% of population at week 24 with/without 1.5% TU-100.

Phylum	Group 1	Group 2	Group 3	*P* value^*∗*^
	(%)	(%)	(%)	(group 1 versus group 2)
Female samples				
Bacteroidetes	28.95 ± 6.66	49.13 ± 10.13	42.02 ± 14.77	0.0012
Deferribacteres	3.30 ± 1.55	3.08 ± 5.94	0.67 ± 0.68	ns
Firmicutes	46.23 ± 6.91	37.37 ± 10.49	45.78 ± 14.46	ns
Proteobacteria	20.13 ± 3.67	8.89 ± 3.02	10.64 ± 2.51	0.0003

Male samples				
Actinobacteria	0.15 ± 0.05	1.74 ± 1.40	1.46 ± 2.33	0.0006
Bacteroidetes	40.39 ± 7.89	36.53 ± 11.15	38.03 ± 9.11	ns
Deferribacteres	2.23 ± 2.87	3.43 ± 4.27	1.62 ± 1.75	ns
Firmicutes	40.99 ± 10.44	41.92 ± 3.64	39.95 ± 7.48	ns
Proteobacteria	14.77 ± 6.19	14.97 ± 9.10	17.73 ± 8.29	ns

^*∗*^Mann–Whitney *U* test.

**Table:** (a) Female samples

Genus level	Group 1	Group 2	Group 3	*P* value^*∗*^
	(%)	(%)	(%)	(group 1 versus group 2)
k__Bacteria;p__Bacteroidetes;c__Bacteroidia;o__Bacteroidales;f__Bacteroidaceae;g__Bacteroides	0.85 ± 0.43	3.53 ± 1.33	2.45 ± 1.71	0.0012
k__Bacteria;p__Bacteroidetes;c__Bacteroidia;o__Bacteroidales;f__Rikenellaceae;g__	1.46 ± 0.89	4.66 ± 1.87	1.44 ± 1.06	0.0012
k__Bacteria;p__Bacteroidetes;c__Bacteroidia;o__Bacteroidales;f__S24-7;g__	14.12 ± 2.51	24.93 ± 5.93	20.13 ± 7.24	0.0093
k__Bacteria;p__Firmicutes;c__Bacilli;o__Turicibacterales;f__Turicibacteraceae;g__Turicibacter	0.16 ± 0.29	1.50 ± 1.37	0.92 ± 1.24	0.0022
k__Bacteria;p__Firmicutes;c__Clostridia;o__Clostridiales;f__;g__	23.34 ± 5.09	14.45 ± 4.46	21.38 ± 13.40	0.0059
k__Bacteria;p__Firmicutes;c__Clostridia;o__Clostridiales;f__Clostridiaceae;g__	0.18 ± 0.07	1.13 ± 0.64	0.62 ± 0.47	0.0003
k__Bacteria;p__Proteobacteria;c__Deltaproteobacteria;o__Desulfovibrionales;f__Desulfovibrionaceae;g__	17.05 ± 4.75	7.35 ± 2.69	8.85 ± 2.19	0.0003
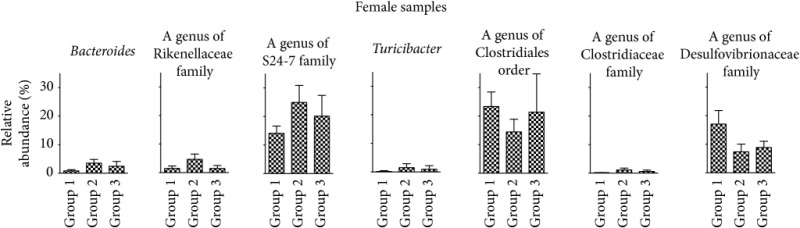				

^*∗*^Mann–Whitney *U* test.

**Table:** (b) Male samples

Genus level	Group 1	Group 2	Group 3	*P* value^*∗*^
	(%)	(%)	(%)	(group 1 versus group 2)
k__Bacteria;p__Actinobacteria;c__Coriobacteriia;o__Coriobacteriales;f__Coriobacteriaceae;g__	0.03 ± 0.02	1.18 ± 1.25	0.77 ± 1.48	0.0006
k__Bacteria;p__Firmicutes;c__Bacilli;o__Turicibacterales;f__Turicibacteraceae;g__Turicibacter	1.82 ± 1.88	0.05 ± 0.08	0.17 ± 0.17	0.0200
k__Bacteria;p__Firmicutes;c__Clostridia;o__Clostridiales;f__Lachnospiraceae;g__[Ruminococcus]	1.47 ± 0.45	0.75 ± 0.19	0.85 ± 0.30	0.0006
k__Bacteria;p__Firmicutes;c__Erysipelotrichi;o__Erysipelotrichales;f__Erysipelotrichaceae;g__Allobaculum	5.33 ± 3.52	17.71 ± 6.29	15.44 ± 6.84	0.0006
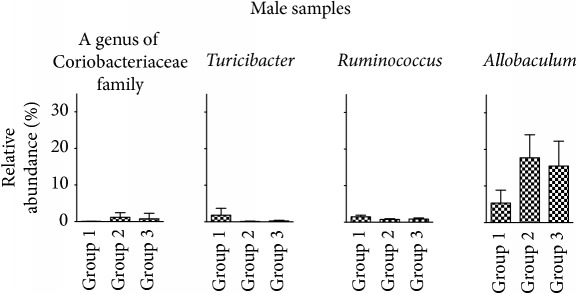				

^*∗*^Mann–Whitney *U* test.

**Table 3:** Phyla with more than 1.0% of population at week 24 with/without 0.75%/3.0% TU-100.

Phylum	Group 1	Group 2	Group 3	Group 4	Group 5	*P* value^*∗*^	*P* value^*∗*^
	(%)	(%)	(%)	(%)	(%)	(group 1 versus group 3)	(group 1 versus group 5)
Actinobacteria	1.17 ± 0.64	0.67 ± 0.57	0.31 ± 0.13	0.31 ± 0.29	0.59 ± 0.53	0.0317	ns
Bacteroidetes	41.58 ± 8.45	39.99 ± 9.61	44.80 ± 6.26	33.43 ± 9.79	45.84 ± 19.47	ns	ns
Deferribacteres	1.17 ± 1.11	1.97 ± 3.31	3.67 ± 4.05	5.01 ± 4.56	6.15 ± 7.07	ns	ns
Firmicutes	42.05 ± 3.17	42.67 ± 3.72	38.41 ± 10.00	39.58 ± 7.33	34.20 ± 7.70	ns	ns
Proteobacteria	13.33 ± 6.69	14.11 ± 5.40	11.8 ± 2.06	20.87 ± 11.30	12.58 ± 8.96	ns	ns

^*∗*^Mann–Whitney *U* test.

**Table 4:** Genera with more than 1.0% of population showing significant alteration by 0.75%/3.0% TU-100 administration for 24 weeks.

Genus level	Group 1	Group 2	Group 3	Group 4	Group 5	*P* value^*∗*^	*P* value^*∗*^
	(%)	(%)	(%)	(%)	(%)	(group 1 versus group 3)	(group 1 versus group 5)
k__Bacteria;p__Actinobacteria;c__Coriobacteriia;o__Coriobacteriales;f__Coriobacteriaceae;g__	1.03 ± 0.56	0.60 ± 0.54	0.08 ± 0.04	0.21 ± 0.26	0.06 ± 0.04	0.0159	0.0159
k__Bacteria;p__Bacteroidetes;c__Bacteroidia;o__Bacteroidales;f__Rikenellaceae;g__	1.19 ± 0.41	0.91 ± 0.72	5.43 ± 2.70	3.36 ± 0.80	3.66 ± 1.95	0.0159	0.0317
k__Bacteria;p__Bacteroidetes;c__Bacteroidia;o__Bacteroidales;f__Rikenellaceae;g__AF12	0.79 ± 0.33	0.74 ± 0.28	1.41 ± 0.32	1.77 ± 0.92	1.34 ± 0.15	ns	0.0317
k__Bacteria;p__Bacteroidetes;c__Bacteroidia;o__Bacteroidales;f__Rikenellaceae;g__Rikenella	0.42 ± 0.33	0.32 ± 0.22	1.30 ± 0.27	1.42 ± 0.64	0.56 ± 0.33	0.0159	ns
k__Bacteria;p__Firmicutes;c__Bacilli;o__Lactobacillales;f__Streptococcaceae;g__Lactococcus	0.59 ± 0.26	0.71 ± 0.55	1.44 ± 0.38	1.29 ± 1.31	0.84 ± 0.70	0.0159	ns
k__Bacteria;p__Firmicutes;c__Clostridia;o__Clostridiales;f__Lachnospiraceae;g__[Ruminococcus]	1.05 ± 0.42	0.67 ± 0.20	0.90 ± 0.64	0.87 ± 0.23	0.54 ± 0.17	ns	0.0317
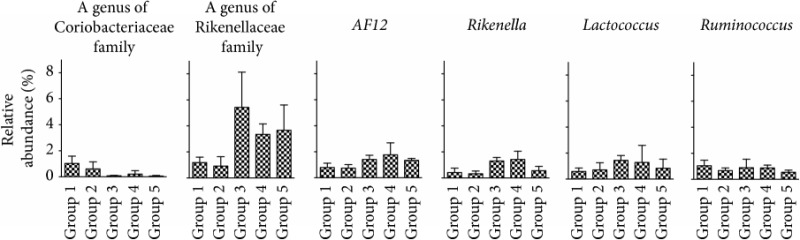							

## Abbreviations

GI: Gastrointestinal tract
QIIME: Quantitative Insights into Microbial Ecology
OTUs: Operational taxonomic units
PCoA: Principal coordinate analysis.

## Additional Points

*Accession Numbers*. The accession numbers for the mouse sample information and the microbial dataset reported in this paper are BioProject: PRJNA403836 and SRA: SRP117766, respectively.

## Supplementary Materials

Supplementary Materials Supplementary Figure  1: the difference in male fecal microbiotas at week 0 between experiments 1 and 2. (A) The PCoA plots of unweighted and weighted UniFrac distances were generated for male fecal microbiotas at week 0 in experiments 1 (red squares) and 2 (blue circles). Both PCoA plots showed the different distribution between experiments. (B) The composition of phyla at week 0 of male samples in both experiments is assigned. Supplementary Table  1: the OTUs with significant increase and decrease by 1.5% TU-100 administration for 24 weeks. Supplementary Table  2: the OTUs with significant increase and decrease by 0.75% TU-100 administration for 24 weeks. Supplementary Table  3: the OTUs with significant increase and decrease by 3.0% TU-100 administration for 24 weeks.
