# Supplementary material for: Time-, Sex-, and Dose-Dependent Alterations of the Gut Microbiota by Consumption of Dietary Daikenchuto (TU-100)
Source: Evid Based Complement Alternat Med. 2018 Feb 22;2018:7415975. doi: 10.1155/2018/7415975 (PMC5842691; doi:10.1155/2018/7415975)
Supplement: Supplementary Materials — Supplementary Figure 1: the difference in male fecal microbiotas at week 0 between experiments 1 and 2. (A) The PCoA plots of unweighted and weighted UniFrac distances were generated for male fecal microbiotas at week 0 in experiments 1 (red squares) and 2 (blue circles). Both PCoA plots showed the different distribution between experiments. (B) The composition of phyla at week 0 of male samples in both experiments is assigned. Supplementary Table 1: the OTUs with significant increase and decrease by 1.5% TU-100 administration for 24 weeks. Supplementary Table 2: the OTUs with significant increase and decrease by 0.75% TU-100 administration for 24 weeks. Supplementary Table 3: the OTUs with significant increase and decrease by 3.0% TU-100 administration for 24 weeks. [file 7415975.f1.pdf]

Supplementary Figure 1

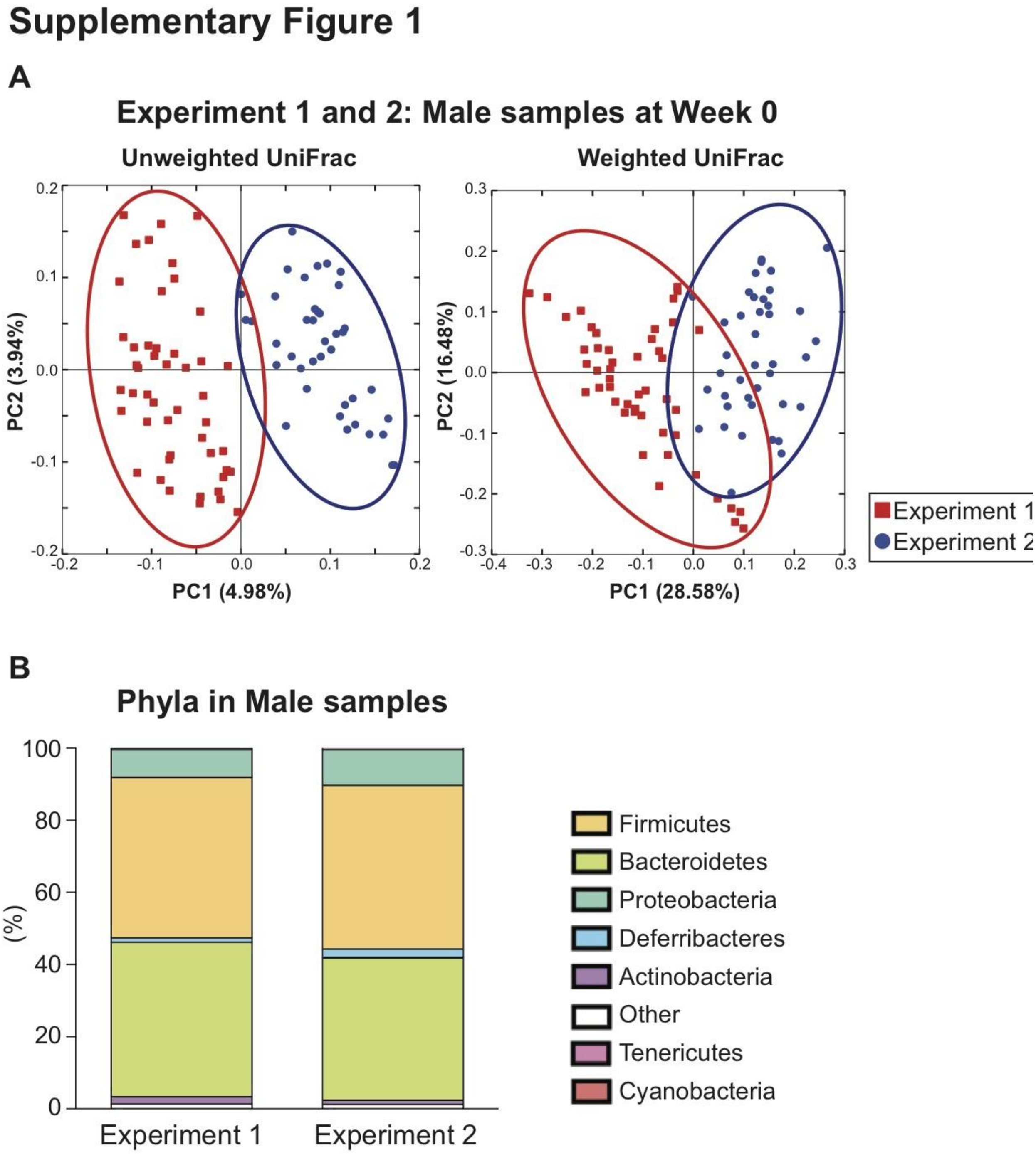

Supplementary Table 1. The OTUs with significant increase and decrease by 1.5% TU-100 administration for 24 weeks

Female samples - Increased OTUs (top 20)

Taxonomy	*P* value* (G1vsG2)
k__Bacteria; p__Firmicutes; c__Clostridia; o__Clostridiales; f__Clostridiaceae; g__; s__	0.000628416
k__Bacteria; p__Firmicutes; c__Clostridia; o__Clostridiales; f__; g__; s__	0.000628416
k__Bacteria; p__Firmicutes; c__Clostridia; o__Clostridiales; f__Clostridiaceae; g__; s__	0.000636299
k__Bacteria; p__Firmicutes; c__Clostridia; o__Clostridiales; f__Lachnospiraceae; g__; s__	0.000817973
k__Bacteria; p__Firmicutes; c__Clostridia; o__Clostridiales; f__Clostridiaceae; g__; s__	0.000956935
k__Bacteria; p__Bacteroidetes; c__Bacteroidia; o__Bacteroidales; f__S24-7; g__; s__	0.001506616
k__Bacteria; p__Bacteroidetes; c__Bacteroidia; o__Bacteroidales; f__Rikenellaceae; g__; s__	0.001677644
k__Bacteria; p__Firmicutes; c__Clostridia; o__Clostridiales; f__; g__; s__	0.001861375
k__Bacteria; p__Firmicutes; c__Clostridia; o__Clostridiales; f__; g__; s__	0.002102622
k__Bacteria; p__Bacteroidetes; c__Bacteroidia; o__Bacteroidales; f__S24-7; g__; s__	0.002981194
k__Bacteria; p__Firmicutes; c__Clostridia; o__Clostridiales; f__; g__; s__	0.003059976
k__Bacteria; p__Firmicutes; c__Clostridia; o__Clostridiales	0.003159791
k__Bacteria; p__Firmicutes; c__Clostridia; o__Clostridiales; f__; g__; s__	0.003351351
k__Bacteria; p__Bacteroidetes; c__Bacteroidia; o__Bacteroidales; f__S24-7; g__; s__	0.003951340
k__Bacteria; p__Bacteroidetes; c__Bacteroidia; o__Bacteroidales; f__Rikenellaceae; g__; s__	0.003963742
k__Bacteria; p__Firmicutes; c__Clostridia; o__Clostridiales; f__; g__; s__	0.004036737
k__Bacteria; p__Bacteroidetes; c__Bacteroidia; o__Bacteroidales; f__S24-7; g__; s__	0.004036737
k__Bacteria; p__Firmicutes; c__Bacilli; o__Turicibacterales; f__Turicibacteraceae; g__Turicibacter; s__	0.004469818
k__Bacteria; p__Bacteroidetes; c__Bacteroidia; o__Bacteroidales; f__S24-7; g__; s__	0.004638759
k__Bacteria; p__Bacteroidetes; c__Bacteroidia; o__Bacteroidales; f__Rikenellaceae; g__; s__	0.005395294

*Kruskal-Wallis test

Female samples - Decreased OTUs (top 20)

Taxonomy	*P* value* (G1vsG2)
k__Bacteria; p__Proteobacteria; c__Deltaproteobacteria; o__Desulfovibrionales; f__Desulfovibrionaceae; g__; s__	0.001054166
k__Bacteria; p__Proteobacteria; c__Deltaproteobacteria; o__Desulfovibrionales; f__Desulfovibrionaceae; g__; s__	0.001193745
k__Bacteria; p__Proteobacteria; c__Deltaproteobacteria; o__Desulfovibrionales; f__Desulfovibrionaceae; g__Desulfovibrio; s__	0.001348349
k__Bacteria; p__Proteobacteria; c__Deltaproteobacteria; o__Desulfovibrionales; f__Desulfovibrionaceae; g__; s__	0.001780211
k__Bacteria; p__Proteobacteria; c__Deltaproteobacteria; o__Desulfovibrionales; f__Desulfovibrionaceae; g__; s__	0.002001649
k__Bacteria; p__Firmicutes; c__Clostridia; o__Clostridiales; f__; g__; s__	0.002629815
k__Bacteria; p__Firmicutes; c__Clostridia; o__Clostridiales; f__Lachnospiraceae; g__; s__	0.003777168
k__Bacteria; p__Firmicutes; c__Clostridia; o__Clostridiales; f__; g__; s__	0.004052727
k__Bacteria; p__Firmicutes; c__Clostridia; o__Clostridiales; f__Lachnospiraceae; g__[Ruminococcus]; s__gnavus	0.005254493
k__Bacteria; p__Firmicutes; c__Clostridia; o__Clostridiales; f__; g__; s__	0.005606455
k__Bacteria; p__Firmicutes; c__Clostridia; o__Clostridiales; f__Ruminococcaceae; g__; s__	0.007719174
k__Bacteria; p__Proteobacteria; c__Deltaproteobacteria; o__Desulfovibrionales; f__Desulfovibrionaceae; g__Desulfovibrio; s__	0.008724379
k__Bacteria; p__Firmicutes; c__Clostridia; o__Clostridiales; f__; g__; s__	0.01181794
k__Bacteria; p__Firmicutes; c__Clostridia; o__Clostridiales; f__Ruminococcaceae; g__Oscillospira; s__	0.014460697
k__Bacteria; p__Proteobacteria; c__Deltaproteobacteria; o__Desulfovibrionales; f__Desulfovibrionaceae; g__; s__	0.016827409
k__Bacteria; p__Firmicutes; c__Clostridia; o__Clostridiales; f__; g__; s__	0.016827409

k__Bacteria; p__Proteobacteria; c__Deltaproteobacteria; o__Desulfovibrionales; f__Desulfovibrionaceae; g__; s__	0.017319383
k__Bacteria; p__Firmicutes; c__Clostridia; o__Clostridiales; f__; g__; s__	0.017571067
k__Bacteria; p__Firmicutes; c__Clostridia; o__Clostridiales; f__Ruminococcaceae; g__; s__	0.018102455
k__Bacteria; p__Bacteroidetes; c__Bacteroidia; o__Bacteroidales; f__S24-7; g__; s__	0.023103394

*Kruskal-Wallis test

Male samples - Increased OTUs (top 20)

Taxonomy	*P* value* (G1vsG2)
k__Bacteria; p__Actinobacteria; c__Coriobacteriia; o__Coriobacteriales; f__Coriobacteriaceae; g__; s__	0.000828873
k__Bacteria; p__Firmicutes; c__Erysipelotrichi; o__Erysipelotrichales; f__Erysipelotrichaceae; g__Allobaculum; s__	0.001455520
k__Bacteria; p__Firmicutes; c__Clostridia; o__Clostridiales; f__; g__; s__	0.001625220
k__Bacteria; p__Bacteroidetes; c__Bacteroidia; o__Bacteroidales; f__Rikenellaceae; g__; s__	0.001644824
k__Bacteria; p__Firmicutes; c__Clostridia; o__Clostridiales; f__; g__; s__	0.001644824
k__Bacteria; p__Firmicutes; c__Erysipelotrichi; o__Erysipelotrichales; f__Erysipelotrichaceae; g__Allobaculum; s__	0.001862165
k__Bacteria; p__Tenericutes; c__Mollicutes; o__Anaeroplasmatales; f__Anaeroplasmataceae; g__Anaeroplasma; s__	0.002108500
k__Bacteria; p__Firmicutes; c__Erysipelotrichi; o__Erysipelotrichales; f__Erysipelotrichaceae; g__Allobaculum; s__	0.002675786
k__Bacteria; p__Firmicutes; c__Erysipelotrichi; o__Erysipelotrichales; f__Erysipelotrichaceae; g__Allobaculum; s__	0.002773451
k__Bacteria; p__Firmicutes; c__Bacilli; o__Lactobacillales; f__Lactobacillaceae; g__Lactobacillus; s__	0.002924386
k__Bacteria; p__Proteobacteria; c__Betaproteobacteria; o__Burkholderiales; f__; g__; s__	0.003092377
k__Bacteria; p__Firmicutes; c__Erysipelotrichi; o__Erysipelotrichales; f__Erysipelotrichaceae; g__Allobaculum; s__	0.003321031
k__Bacteria; p__Firmicutes; c__Erysipelotrichi; o__Erysipelotrichales; f__Erysipelotrichaceae; g__Allobaculum; s__	0.005948559
k__Bacteria; p__Firmicutes; c__Erysipelotrichi; o__Erysipelotrichales; f__Erysipelotrichaceae; g__Allobaculum; s__	0.007643524
k__Bacteria; p__Firmicutes; c__Erysipelotrichi; o__Erysipelotrichales; f__Erysipelotrichaceae; g__Allobaculum; s__	0.008150972
k__Bacteria; p__Firmicutes; c__Erysipelotrichi; o__Erysipelotrichales; f__Erysipelotrichaceae; g__Allobaculum; s__	0.008563666
k__Bacteria; p__Bacteroidetes; c__Bacteroidia; o__Bacteroidales; f__Prevotellaceae; g__Prevotella; s__	0.009057888
k__Bacteria; p__Proteobacteria; c__Deltaproteobacteria; o__Desulfovibrionales; f__Desulfovibrionaceae; g__Desulfovibrio; s__	0.009600749
k__Bacteria; p__Firmicutes; c__Erysipelotrichi; o__Erysipelotrichales; f__Erysipelotrichaceae; g__Allobaculum; s__	0.010555302
Unassigned	0.011410283

*Kruskal-Wallis test

Male samples - Decreased OTUs (top 20)

Taxonomy	*P* value* (G1vsG2)
Unassigned	0.001045853
k__Bacteria; p__Firmicutes; c__Clostridia; o__Clostridiales; f__; g__; s__	0.001644824
k__Bacteria; p__Bacteroidetes; c__Bacteroidia; o__Bacteroidales; f__S24-7; g__; s__	0.001745119
k__Bacteria; p__Firmicutes; c__Clostridia; o__Clostridiales; f__Lachnospiraceae; g__[Ruminococcus]; s__gnavus	0.003670617
k__Bacteria; p__Firmicutes; c__Clostridia; o__Clostridiales; f__Ruminococcaceae; g__Oscillospira; s__	0.007200651
k__Bacteria; p__Firmicutes; c__Clostridia; o__Clostridiales; f__; g__; s__	0.008010122
k__Bacteria; p__Bacteroidetes; c__Bacteroidia; o__Bacteroidales; f__S24-7; g__; s__	0.008150972
k__Bacteria; p__Firmicutes; c__Clostridia; o__Clostridiales; f__; g__; s__	0.008750216
k__Bacteria; p__Firmicutes; c__Clostridia; o__Clostridiales; f__Clostridiaceae	0.008750216
k__Bacteria; p__Firmicutes; c__Clostridia; o__Clostridiales; f__Lachnospiraceae; g__; s__	0.009161627
k__Bacteria; p__Firmicutes; c__Clostridia; o__Clostridiales; f__; g__; s__	0.009596760
k__Bacteria; p__Tenericutes; c__Mollicutes; o__Mycoplasmatales; f__Mycoplasmataceae; g__; s__	0.013688169
k__Bacteria; p__Firmicutes; c__Clostridia; o__Clostridiales; f__; g__; s__	0.014529532

k__Bacteria; p__Firmicutes; c__Bacilli; o__Turicibacterales; f__Turicibacteraceae; g__Turicibacter; s__	0.015624363
k__Bacteria; p__Bacteroidetes; c__Bacteroidia; o__Bacteroidales; f__S24-7; g__; s__	0.017833566
k__Bacteria; p__Firmicutes; c__Clostridia; o__Clostridiales; f__Ruminococcaceae; g__; s__	0.019206777
k__Bacteria; p__Firmicutes; c__Clostridia; o__Clostridiales; f__Ruminococcaceae; g__Oscillospira; s__	0.022586888
k__Bacteria; p__Firmicutes; c__Clostridia; o__Clostridiales; f__; g__; s__	0.022947809
k__Bacteria; p__Firmicutes; c__Clostridia; o__Clostridiales; f__; g__; s__	0.023755991
k__Bacteria; p__Firmicutes; c__Clostridia; o__Clostridiales; f__Ruminococcaceae; g__Ruminococcus; s__	0.024547111

*Kruskal-Wallis test

Supplementary Table 2. The OTUs with significant increase and decrease by 0.75% TU-100 administration for 24 weeks

0.75% TU-100 for 24 weeks - Increased OTUs (top 20)

Taxonomy	*P* value* (G1vsG3)
k__Bacteria; p__Firmicutes; c__Bacilli; o__Lactobacillales; f__Lactobacillaceae; g__Lactobacillus; s__	0.00671770
k__Bacteria; p__Firmicutes; c__Clostridia; o__Clostridiales; f__; g__; s__	0.00671770
k__Bacteria; p__Firmicutes; c__Clostridia; o__Clostridiales; f__Lachnospiraceae; g__; s__	0.00700094
k__Bacteria; p__Firmicutes; c__Clostridia; o__Clostridiales; f__; g__; s__	0.00729036
k__Bacteria; p__Firmicutes; c__Clostridia; o__Clostridiales; f__Lachnospiraceae; g__; s__	0.00729036
k__Bacteria; p__Firmicutes; c__Clostridia; o__Clostridiales; f__; g__; s__	0.00729036
k__Bacteria; p__Firmicutes; c__Clostridia; o__Clostridiales; f__Ruminococcaceae; g__Oscillospira; s__	0.00729036
k__Bacteria; p__Proteobacteria; c__Epsilonproteobacteria; o__Campylobacterales; f__Helicobacteraceae; g__Helicobacter; s__	0.01051525
k__Bacteria; p__Bacteroidetes; c__Bacteroidia; o__Bacteroidales; f__S24-7; g__; s__	0.01086976
k__Bacteria; p__Firmicutes; c__Clostridia; o__Clostridiales; f__Ruminococcaceae; g__Oscillospira; s__	0.01311266
k__Bacteria; p__Bacteroidetes; c__Bacteroidia; o__Bacteroidales; f__Rikenellaceae; g__; s__	0.01390299
k__Bacteria; p__Firmicutes; c__Clostridia; o__Clostridiales; f__; g__; s__	0.01417139
k__Bacteria; p__Firmicutes; c__Bacilli; o__Lactobacillales; f__Streptococcaceae; g__Lactococcus; s__	0.01430588
k__Bacteria; p__Bacteroidetes; c__Bacteroidia; o__Bacteroidales; f__Rikenellaceae; g__; s__	0.01430588
k__Bacteria; p__Bacteroidetes; c__Bacteroidia; o__Bacteroidales; f__S24-7; g__; s__	0.01430588
k__Bacteria; p__Bacteroidetes; c__Bacteroidia; o__Bacteroidales; f__Rikenellaceae; g__Rikenella; s__	0.01430588
k__Bacteria; p__Bacteroidetes; c__Bacteroidia; o__Bacteroidales; f__S24-7; g__; s__	0.01648450
k__Bacteria; p__Firmicutes; c__Clostridia; o__Clostridiales; f__Lachnospiraceae; g__[Ruminococcus]; s__gnavus	0.01899597
k__Bacteria; p__Firmicutes; c__Clostridia; o__Clostridiales; f__; g__; s__	0.01899597
k__Bacteria; p__Bacteroidetes; c__Bacteroidia; o__Bacteroidales; f__S24-7; g__; s__	0.02365162

*Kruskal-Wallis test

0.75% TU-100 for 24 weeks - Decreased OTUs (all of 15 OTUs)

Taxonomy	*P* value* (G1vsG3)
k__Bacteria; p__Actinobacteria; c__Coriobacteriia; o__Coriobacteriales; f__Coriobacteriaceae; g__; s__	0.01051525
k__Bacteria; p__Firmicutes; c__Clostridia; o__Clostridiales; f__; g__; s__	0.01122989
k__Bacteria; p__Firmicutes; c__Clostridia; o__Clostridiales; f__Ruminococcaceae; g__Ruminococcus; s__	0.01311266
k__Bacteria; p__Firmicutes; c__Clostridia; o__Clostridiales; f__Ruminococcaceae; g__Oscillospira; s__	0.01390299
k__Bacteria; p__Firmicutes; c__Clostridia; o__Clostridiales; f__; g__; s__	0.01430588
k__Bacteria; p__Firmicutes; c__Clostridia; o__Clostridiales; f__; g__; s__	0.01745104
k__Bacteria; p__Bacteroidetes; c__Bacteroidia; o__Bacteroidales; f__S24-7; g__; s__	0.01945103
k__Bacteria; p__Firmicutes; c__Clostridia; o__Clostridiales; f__; g__; s__	0.0218957
k__Bacteria; p__Firmicutes; c__Clostridia; o__Clostridiales; f__Ruminococcaceae	0.02365162
k__Bacteria; p__Firmicutes; c__Erysipelotrichi; o__Erysipelotrichales; f__Erysipelotrichaceae; g__Allobaculum; s__	0.03012682
k__Bacteria; p__Bacteroidetes; c__Bacteroidia; o__Bacteroidales; f__S24-7; g__; s__	0.03012682
k__Bacteria; p__Firmicutes; c__Clostridia; o__Clostridiales; f__Lachnospiraceae; g__[Ruminococcus]; s__gnavus	0.03576081
k__Bacteria; p__Firmicutes; c__Clostridia; o__Clostridiales; f__Lachnospiraceae; g__[Ruminococcus]; s__gnavus	0.03576081
k__Bacteria; p__Firmicutes; c__Clostridia; o__Clostridiales; f__; g__; s__	0.0388671
k__Bacteria; p__Proteobacteria; c__Epsilonproteobacteria; o__Campylobacterales; f__Helicobacteraceae; g__Helicobacter; s__	0.04909012

*Kruskal-Wallis test

Supplementary Table 3. The OTUs with significant increase and decrease by 3.0% TU-100 administration for 24 weeks

3.0% TU-100 for 24 weeks - Increased OTUs (top 20)

Taxonomy	*P* value* (G1vsG5)
k__Bacteria; p__Bacteroidetes; c__Bacteroidia; o__Bacteroidales; f__Bacteroidaceae; g__Bacteroides; s__	0.00467773
k__Bacteria; p__Firmicutes; c__Clostridia; o__Clostridiales; f__; g__; s__	0.00616990
k__Bacteria; p__Firmicutes; c__Clostridia; o__Clostridiales; f__Ruminococcaceae; g__Ruminococcus; s__	0.00700094
k__Bacteria; p__Firmicutes; c__Clostridia; o__Clostridiales; f__; g__; s__	0.00700094
k__Bacteria; p__Firmicutes; c__Clostridia; o__Clostridiales; f__; g__; s__	0.00700094
k__Bacteria; p__Firmicutes; c__Clostridia; o__Clostridiales; f__; g__; s__	0.00700094
k__Bacteria; p__Bacteroidetes; c__Bacteroidia; o__Bacteroidales; f__Bacteroidaceae; g__Bacteroides; s__	0.00700094
k__Bacteria; p__Bacteroidetes; c__Bacteroidia; o__Bacteroidales; f__S24-7; g__; s__	0.00729036
k__Bacteria; p__Firmicutes; c__Clostridia; o__Clostridiales; f__Ruminococcaceae; g__Oscillospira; s__	0.00729036
k__Bacteria; p__Bacteroidetes; c__Bacteroidia; o__Bacteroidales; f__Rikenellaceae; g__; s__	0.00729036
k__Bacteria; p__Firmicutes; c__Clostridia; o__Clostridiales; f__; g__; s__	0.01016640
k__Bacteria; p__Bacteroidetes; c__Bacteroidia; o__Bacteroidales; f__Rikenellaceae; g__; s__	0.01051525
k__Bacteria; p__Firmicutes; c__Clostridia; o__Clostridiales; f__; g__; s__	0.01051525
k__Bacteria; p__Firmicutes; c__Clostridia; o__Clostridiales; f__; g__; s__	0.01051525
k__Bacteria; p__Firmicutes; c__Clostridia; o__Clostridiales; f__; g__; s__	0.01051525
k__Bacteria; p__Bacteroidetes; c__Bacteroidia; o__Bacteroidales; f__S24-7; g__; s__	0.01051525
k__Bacteria; p__Bacteroidetes; c__Bacteroidia; o__Bacteroidales; f__Rikenellaceae; g__; s__	0.01234336
k__Bacteria; p__Bacteroidetes; c__Bacteroidia; o__Bacteroidales; f__S24-7; g__; s__	0.01350523
k__Bacteria; p__Bacteroidetes; c__Bacteroidia; o__Bacteroidales; f__S24-7; g__; s__	0.01390299
k__Bacteria; p__Bacteroidetes; c__Bacteroidia; o__Bacteroidales; f__Bacteroidaceae; g__Bacteroides; s__	0.01430588

*Kruskal-Wallis test

3.0% TU-100 for 24 weeks - Decreased OTUs (top 20)

Taxonomy	*P* value* (G1vsG5)
k__Bacteria; p__Firmicutes; c__Clostridia; o__Clostridiales; f__; g__; s__	0.00915440
k__Bacteria; p__Firmicutes; c__Clostridia; o__Clostridiales; f__; g__; s__	0.00982327
k__Bacteria; p__Actinobacteria; c__Coriobacteriia; o__Coriobacteriales; f__Coriobacteriaceae; g__; s__	0.01051525
k__Bacteria; p__Bacteroidetes; c__Bacteroidia; o__Bacteroidales; f__[Odoribacteraceae]; g__Odoribacter; s__	0.01051525
k__Bacteria; p__Firmicutes; c__Clostridia; o__Clostridiales; f__; g__; s__	0.01272535
k__Bacteria; p__Firmicutes; c__Erysipelotrichi; o__Erysipelotrichales; f__Erysipelotrichaceae; g__Allobaculum; s__	0.01272535
k__Bacteria; p__Firmicutes; c__Clostridia; o__Clostridiales; f__Lachnospiraceae; g__[Ruminococcus]; s__gnavus	0.01390299
k__Bacteria; p__Bacteroidetes; c__Bacteroidia; o__Bacteroidales; f__S24-7; g__; s__	0.01430588
k__Bacteria; p__Firmicutes; c__Clostridia; o__Clostridiales; f__; g__; s__	0.01696491
k__Bacteria; p__Proteobacteria; c__Deltaproteobacteria; o__Desulfovibrionales; f__Desulfovibrionaceae; g__Desulfovibrio; s__	0.02494664
k__Bacteria; p__Firmicutes; c__Clostridia; o__Clostridiales; f__; g__; s__	0.02620604
k__Bacteria; p__Bacteroidetes; c__Bacteroidia; o__Bacteroidales; f__Porphyromonadaceae; g__Parabacteroides; s__	0.02748634
k__Bacteria; p__Bacteroidetes; c__Bacteroidia; o__Bacteroidales; f__S24-7; g__; s__	0.02748634
k__Bacteria; p__Bacteroidetes; c__Bacteroidia; o__Bacteroidales; f__S24-7; g__; s__	0.02845974
k__Bacteria; p__Bacteroidetes; c__Bacteroidia; o__Bacteroidales; f__S24-7; g__; s__	0.02845974
k__Bacteria; p__Proteobacteria; c__Deltaproteobacteria; o__Desulfovibrionales; f__Desulfovibrionaceae; g__; s__	0.02845974

k__Bacteria; p__Bacteroidetes; c__Bacteroidia; o__Bacteroidales; f__S24-7; g__; s__	0.02845974
k__Bacteria; p__Firmicutes; c__Erysipelotrichi; o__Erysipelotrichales; f__Erysipelotrichaceae; g__Allobaculum; s__	0.02845974
k__Bacteria; p__Firmicutes; c__Clostridia; o__Clostridiales; f__; g__; s__	0.03097143
k__Bacteria; p__Bacteroidetes; c__Bacteroidia; o__Bacteroidales; f__S24-7; g__; s__	0.03097143

*Kruskal-Wallis test
